# Deep phenotyping platform for microscopic plant-pathogen interactions

**DOI:** 10.3389/fpls.2025.1462694

**Published:** 2025-02-03

**Authors:** Stefanie Lück, Salim Bourras, Dimitar Douchkov

**Affiliations:** ^1^ Department of Breeding Research, Leibniz Institute of Plant Genetics and Crop Plant Research (IPK), Seeland, Germany; ^2^ Department of Plant Biology, Swedish University of Agricultural Sciences (SLU), Uppsala, Sweden

**Keywords:** BluVision, automated microscopy, barley, deep learning, microphenomics, neuronal networks, pathogens, powdery mildew

## Abstract

The increasing availability of genetic and genomic resources has underscored the need for automated microscopic phenotyping in plant-pathogen interactions to identify genes involved in disease resistance. Building on accumulated experience and leveraging automated microscopy and software, we developed *BluVision Micro*, a modular, machine learning-aided system designed for high-throughput microscopic phenotyping. This system is adaptable to various image data types and extendable with modules for additional phenotypes and pathogens. *BluVision Micro* was applied to screen 196 genetically diverse barley genotypes for interactions with powdery mildew fungi, delivering accurate, sensitive, and reproducible results. This enabled the identification of novel genetic loci and marker-trait associations in the barley genome. The system also facilitated high-throughput studies of labor-intensive phenotypes, such as precise colony area measurement. Additionally, *BluVision*’s open-source software supports the development of specific modules for various microscopic phenotypes, including high-throughput transfection assays for disease resistance-related genes.

## 1 Introduction

One of the most sustainable and environmentally friendly alternatives to chemical pesticides is harnessing the natural disease resistance of plants. This approach has a long history of success in crop breeding. However, to address new challenges, plant breeders need to discover new sources of disease resistance by exploring the genetic diversity stored in gene banks and germplasm collections worldwide. This requires more sensitive phenotyping tools capable of identifying quantitative trait loci (QTLs) with minimal effects and low allele frequency.

Recognizing this need, the scientific community has developed precise and high-throughput phenotyping tools, establishing a new scientific discipline called phenomics. Most of these efforts have focused on phenotyping at the level of whole plants and canopies, lacking the spatial resolution necessary for detailed studies of microscopic plant-pathogen interactions. To address this gap, we have developed a highly automated phenotyping platform that covers the subcellular, tissue, and organ levels. Our system for organ-level phenotyping on a macroscopic scale, called Macrobot, and the corresponding software framework (*BluVision Macro*), were previously published (Lück et al., 2020; Lueck et al., 2020).

The first software implementation to detect and quantify microcolonies of *B. graminis* on barley and wheat was *HyphArea* (Seiffert and Schweizer, 2005; Baum et al., 2011). The tool pioneered establishing a high-throughput platform for plant-pathogen interaction phenotyping on a microscopic level and allowed access to novel phenotypes, such as quantifying the area of fungal secondary hyphae. However, the high sensitivity and specificity levels of the *HyphArea* Tool demonstrated in (Seiffert and Schweizer, 2005; Baum et al., 2011) were often difficult to reach due to the variability of the sample properties and quality. Besides the image analysis, the extended use of the *HyphArea* revealed issues with the handling and processing of the raw data. The acquired image data were exported as individual camera frames (tiles) and stored in separate TIFF files. This step simplifies image data processing and avoids using proprietary file formats, but it results in a massive expansion of the file number (>10^6^ files for a large screen), thus approaching the limits of the commonly used hardware and software. Finally, the long run time of the *HyphArea* renders it less appropriate for high-throughput phenotyping screenings.

Despite its limitations, *HyphArea* demonstrated the transformative potential of automated microscopy and image analysis in plant-pathogen phenotyping, paving the way for the development of a new software system, *BluVision*, which is presented in this study.

**Figure 1:**
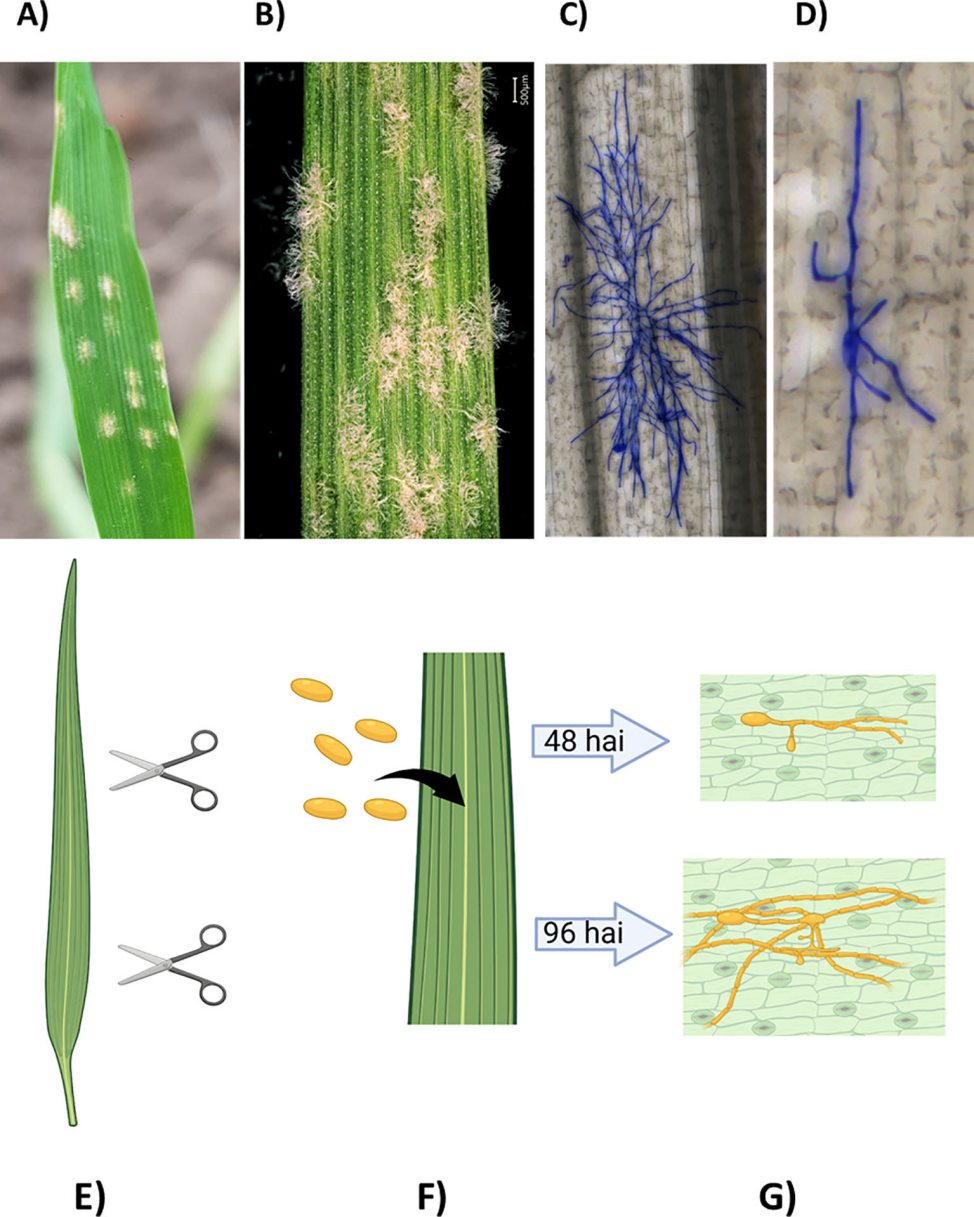
**(A-D)** Visible and microscopic infection phenotypes of powdery mildew on barley. **(A)** Barley leaf with powdery mildew approximately 7 days after infection (dai). **(B)** Barley powdery mildew at higher magnification (6-7 dai). **(C)** Barley powdery mildew on barley 96 hours after infection (hai). **(D)** Barley powdery mildew on barley, 48 hai. **(E-G)** Experimental design. **(E)** Leaf segments are cut from the second leaf of 14-day-old plants. **(F)** The leaf segments are inoculated with fungal spores. **(G)** The samples are collected at different time points after inoculation (e.g., 48, 72, 96 hai) and stained for microscopic analysis.

The primary aim of the *BluVision* framework is to phenotype plant-pathogen interactions on both microscopic and macroscopic levels. We selected the well-established system of the powdery mildew fungus *Blumeria graminis* f.sp. *hordei* (Bgh), a pathogen of barley, as our model (Panstruga and Dodds, 2009; Spanu and Kamper, 2010; Douchkov et al., 2014a, b). *B. graminis* is a species of the Ascomycete genus *Blumeria* in the order *Erysiphales*, causing powdery mildew diseases on various grass species. *Blumeria graminis* are obligate parasites with highly specific host-specialization forms, such as *B. graminis* f. sp. *tritici* (Bgt; wheat powdery mildew) and *B. graminis* f. sp. *hordei* (Bgh; barley powdery mildew) (Wyand and Brown, 2003) (**Figures 1A–D**).

The barley powdery mildew model offers several advantages: (i) the fungus proliferates rapidly and in a highly synchronized manner, (ii) the majority of its biomass is located on the leaf surface, and (iii) it interacts only with the uppermost layer of plant leaf cells, (i.e., the epidermis) via a specialized intracellular feeding organ called a haustorium (Huckelhoven and Panstruga, 2011). This system’s reduced complexity provides an excellent environment for studying plant-pathogen interactions on a microscopic scale. Full-size and multilevel microscopy images of large objects, such as leaf segments, generate complex data sets that have been challenging to analyze with automated image analysis methods until recently. The advent of machine learning (ML) has significantly improved this situation. ML methods use analytical models to identify patterns and make decisions with minimal human intervention (Mitchell, 1997; Voulodimos et al., 2018). There are two main ML approaches: supervised learning from pre-labeled data (Norving and Russell, 2010) and unsupervised learning from unlabeled data (Hinton and Sejnowski, 1999). Image analysis typically involves classification and segmentation steps. Here, features (variables) from images are used to classify objects, while image segmentation assigns labels to individual pixels, grouping them into subgroups (image objects) and separating them from the background (Stockman and Shapiro, 2001). The success of image analysis often depends on choosing meaningful classification features (Zheng and Casari, 2018). This work compares two main methods: manually selecting features (handcrafted) and automatically extracting features using a convolutional neural network (CNN). CNNs can automatically select many features, leading to more robust prediction models, but they require large training datasets. In contrast, predictive models like Random Forest (RF) with carefully selected handcrafted features can perform well even on small training sets (Lin et al., 2020). Thus, the optimal approach depends on the specific application and typically requires preliminary testing of different methods.

Here, we present the *BluVision Micro* system, a novel platform dedicated to phenotyping the initial stages of plant-pathogen interactions using high-throughput automated microscopy and computer vision methods. The aims of this study are threefold: (i) to develop an improved phenotyping tool for detecting and quantifying fungal colonies during early infection stages, building upon and addressing the limitations of the HyphArea tool; (ii) to validate the performance and accuracy of the *BluVision Micro* system; and (iii) to demonstrate the utility of the system by applying it in a genome-wide association study (GWAS) on barley powdery mildew. This comprehensive approach underscores the potential of *BluVision Micro* to advance high-throughput phenotyping for plant-pathogen interactions.

## 2 Material, methods and equipment

### 2.1 Plant and fungal material

Barley cv. Golden Promise and cv. Morex, and wheat cv. Kanzler were grown in 12 cm pots with nursery soil substrate. The plants were incubated in a plant growth cabinet (Panasonic MLR-352H-PE Versatile Environmental Test Chamber, white LED upgrade; Panasonic Healthcare Co., Ltd.) at controlled conditions (dark period of 8h, light period of 16h, 20°C and 60 RH%) for 7 days or 14 days. The first or the second leaves were harvested and mounted on 1% water agar (Phyto agar, Duchefa, the Netherlands) plates supplemented by 20 mg/L benzimidazole (Sigma-Aldrich, the USA) as a senescence inhibitor. The barley leaf segments were inoculated with the Bgh isolate CH4.8, and the wheat leaf segments were inoculated with the Bgt isolate FAL92315 at approximately five spores/mm^2^ in an inoculation tower. The infection was stopped at 36-96 hours after inoculation (hai) by incubating the leaf segments in a clearing solution (7 mL 96% ethanol and 1 mL acetic acid) for 48 hours at room temperature. After that, the fungal colonies were stained with Coomassie staining solution (0.3% Coomassie R250, 7.5% (w/v) trichloroacetic acid, and 50% (v/v) methanol) for 5 minutes and then washed several times with water. The prepared samples were mounted on microscope slides with 50% glycerol to avoid drying the leaves during image acquisition.

A diversity set of 200 barley accessions (BRIDGE Core 200 collection) from the Federal *ex-situ* Genebank in Gatersleben, selected for maximized genetic diversity, were genotyped by using whole-genome sequencing (WGS) data from Illumina short-read sequencing with 3x genome coverage (Milner et al., 2019), and aligned to the barley MorexV2 reference genome (König et al., 2020; Mascher, 2020). A quality filter on 223 387 147 variants was applied with the *PLINK* 2.0 software, limiting the missing values to ≤ 0.02 and minor allele frequency (MAF) to ≥0.05. After filtering, 949 174 high-quality variants remained and were used in GWAS analysis. Four genotypes were eliminated from the set for technical reasons (e.g., poor germination), so the final genotype set consisted of 196 accessions.

The material of the barley core collection of genotypes was grown, collected, and inoculated as described in (Lück et al., 2020). In brief, the plants were grown in 24-well seedling trays with nursery soil substrate, ten plants of the same genotype per well, in a climatized greenhouse (~20°C day/~16°C night) for 14 days. Leaf fragments from the second leaf were harvested and mounted on standard 4-well microtiter plates filled with 1% water agar supplemented by 20 mg/L benzimidazole. The leaf fragments were inoculated, incubated, and stained as described above.

The experiment was conducted in three independent biological repetitions, where plants were grown in three separate batches, and each batch was infected with spores produced at different time points. Within each biological repetition, up to eight technical replicates were included to ensure the robustness and reproducibility of the results.

### 2.2 Image acquisition and analysis hardware

The microscopy image data was acquired on a commercial *Zeiss AxioScan.Z1* high-performance microscopy slide scanner, and ZEN 3.0 (blue edition) software (Carl Zeiss AG). The imaging was done in a bright field configuration with a *Hitachi HV-F202SCL* camera (3 CCD 1/1.8” progressive scan color sensor with 1600x1200 effective pixels and 24-bit color depth), 1x camera adapter. The scanning objective typically used an *EC Plan-Neofluar* 5x/0.16 M27 with 0.16 NA (numerical aperture) that provides a large depth of field (DoF), which was particularly advantageous for scanning very thick and uneven objects as whole-leaf fragments. The acquired image data was stored in a CZI file container that combines all relevant image and meta information in one file. The image data were analyzed on a Windows 10 Enterprise server with a dual *Intel Xeon*™ *E5-2695* processor with 36 physical cores and 512 GB RAM, allowing near real-time analysis.

### 2.3 Software implementation

The software *BluVision Micro* and all experiments were implemented in *Python 3.6* under *Windows 10* operating system. The following free *Python* libraries were used for development: *OpenCV-Python, NumPy, Pandas, Keras, Tensorflow, czifile, skimage, mahotas, joblib* and *Scikit-learn.* Training of the CNN model was done on an *NVIDIA TITAN X GPU* with *Keras* 2.3.1 and *Tensorflow* 2.1.0 backend, and training time of about 20,000 images per hour on an Intel^®^ Core™ i7-9700 CPU 3.00 GHz with 64-Bit Windows 10 operation system.

The software is implemented as a two-step command-line tool with separated image processing and data analysis, allowing curation of the intermediate results without rerunning the entire analysis. In addition, the image processing can be parallelized, depending on the installed computer memory.

### 2.4 Downstream analysis

#### 2.4.1 Genome-wide association scan

GWAS for all traits was conducted using the software tool GWAStic (Lück et al., 2024). Briefly, the tool uses the Factored Spectrally Transformed Linear Mixed Model with a kinship (K) matrix provided by the *FaST-LMM* program (*fastlmm* 0.6.7) (Lippert et al., 2011; Listgarten et al., 2012). A suggestive threshold (−log10 P ≥ 6.0) was calculated based on the formula -log10 (1/number of independent SNPs) (Yang et al., 2014), and a significance threshold (−log10 P ≥ 8.0) for the identification of QTLs was calculated by using the Bonferroni correction method (Hommel, 1988).

#### 2.4.2 Haplotype blocks and linkage disequilibrium analysis

The *PLINK* ‘clumping’ algorithm was employed to choose the most significant SNPs (−log10 P ≥ 5.0, clump-p1 parameter) and locate all SNPs in linkage disequilibrium (LD) (using a clump-r2 parameter set to 0.6). To perform the clumping process, we set the physical distance threshold to 10,000 (clump-kb parameter). In cases where these regions overlapped, we consolidated them into a unified and expanded locus.

#### 2.4.3 Protein domain overrepresentation analysis

Protein domain enrichment analysis was performed using the *ShinyGO* 0.80 online tool (Ge et al., 2020), using the *Morex* V2 gene ID against the *hvtritex_eg_gene* gene database (*Morex* V2 TRITEX assembly) and *GO Molecular Functions* pathway database.

## 3 Software development

### 3.1 Image processing

#### 3.1.1 Focus stacking

**Figure 2:**
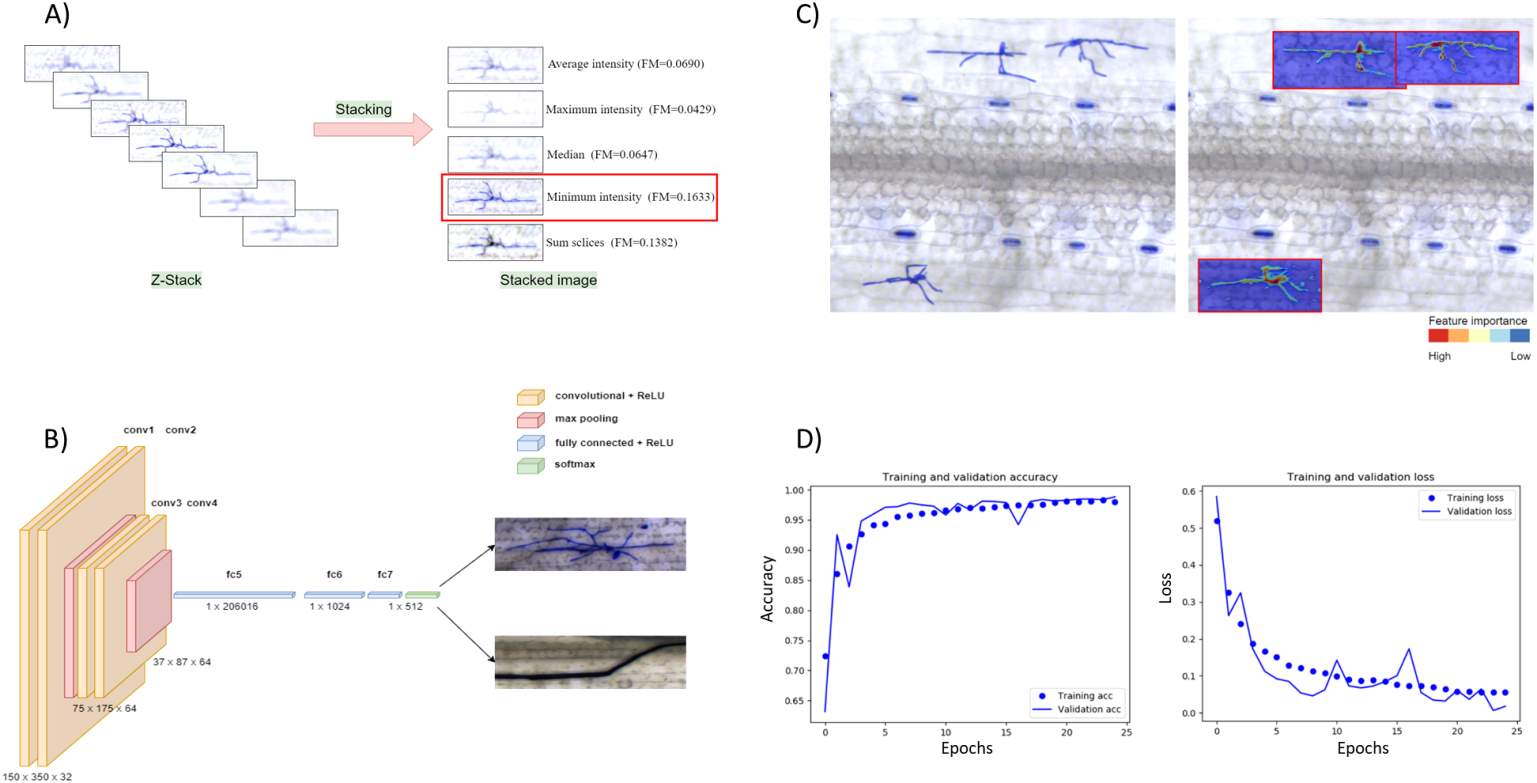
Image processing and model development. **(A)** Comparing stacking algorithms. Five stacking algorithms were compared: *Average intensity, Maximum intensity, Median, Minimum intensity, Sum slices*. The *Minimum intensity* method achieved the highest quality measure (FM). **(B)** The structure of a convolutional neural network consists of convolutional, pooling, and fully connected layers. **(C)** Heatmap visualization shows the significance of the selected features in predicting a target variable (fungal structure). The left image represents the raw image data, and on the right are the regions of interest detected by the software (red border rectangle) with hyphae segmentation. The example clearly shows that the CNN model localizes the fungal colony with high probability (red colors), as the probability in the background drops significantly (blue colors). **(D)** Training and validation accuracy of the CNN model trained with ca. 10,000 positive images.

The first step addresses the problem of capturing all image details of samples whose thickness exceeds the depth of focus of a single image frame. This issue is commonly known as ‘focus stacking’, which is particularly challenging when the target object can be located at different depths (different Z positions) on the samples. In the case of mildew microcolonies, this is typically due to the three-dimensional structure resulting from irregular/interlaced hyphae combined with the leaf surface topology. We tested five different Z-projection methods included in the *Fiji* distribution package of *ImageJ* v1.53 (Schindelin et al., 2012) based on the values of the pixels along a single Z-axis point, namely *Average intensity* (Khamfongkhruea et al., 2017), *Maximum intensity* (Sato et al., 1998), *Minimum intensity* (Hayabuchi et al., 2011), *Sum slices*, *Standard deviation* and *Median* (**Figure 2A**).

Furthermore, for each stacked image, the image Quality Measure (FM) has been computed and compared (**Supplementary Table S19**) (De and Masilamani, 2013). The minimum intensity projection method achieved the best FM score in all tested cases and was selected for the image processing pipeline.

#### 3.1.2 Colony segmentation

The second step in the image processing pipeline focuses on defining the optimal image segmentation strategy to accurately distinguish colony boundaries from the background, referred to as ‘colony segmentation’. A major challenge was designing a reliable pipeline that could tolerate variations in staining quality and background without missing positive objects, such as genuine colonies.

First, Z-stacked images from the initial step were segmented using the *scikit-image* library in *Python*. The raw RGB images were converted to the more informative YQ1Q2 color space, and automatic multilevel thresholding (Yen) was applied to identify the putative regions of interest (ROIs). These ROIs were extracted as bounding boxes. Using the *OpenCV Python* library and the border-following algorithm, contours (the lines connecting all contiguous points sharing the same color or intensity) were identified, and a virtual rectangle delineating each object was drawn. The bounding boxes were then classified as either positive or negative, indicating the presence or absence of a colony ‘object’, respectively.

The use of ‘color space’, a normalized organization of colors supported by various image capture devices, proved valuable for providing reproducible scoring of color across variable staining intensities and qualities within and between ROIs. Different common color spaces were tested: HSV, L*a*b, YCbCr, XYZ, AC1C2, YUV, I1I2I3 and YQ1Q2, in combination with varying algorithms of thresholding: *Yen’s maximum correlation* (Yen et al., 1995), *Li’s minimum cross-entropy* method (Li and Lee, 1993; Li and Tam, 1998; Sezgin and Sankur, 2004), *Otsu* (Otsu, 1979), *Isodata* (Ridler and Calvard, 1978), *Mean* (Glasbey, 1993), *Minimum* (Prewitt and Mendelsohn, 1966; Glasbey, 1993), *Triangle* (Zack et al., 1977), *Canny edge detector* (Canny, 1986) (**Supplementary Table S20**). Combining the Q2 channel from the *YQ1Q2* color space with *Yen’s thresholding* generated the most reliable results. Using only a single-color channel, we achieved a robust and reliable segmentation method that is insensitive to staining variations and performs well on different sizes of the hyphae (36 to 96 hai).

A morphological closing operation was applied to the segmented binary images to close the gaps that may lead to partial object extraction. Finally, a *Moore-Neighbour tracing* algorithm (Weisstein, 2021) was used to extract the contours of the binary image for colony classification.

### 3.2 Machine learning

#### 3.2.1 Training data set

The next challenge was to extract colony features that could be used for phenotyping. Due to the morphological variability in fungal micro-colonies and colony ROIs, each image is unique, making ‘one-size-fits-all’ strategies like thresholding or background subtraction ineffective. We argue that this is a typical problem suited for machine learning (ML).

Developing effective ML models requires generating a high-quality, precise training dataset to help artificial intelligence (AI) algorithms recognize, score, and analyze objects and patterns.

In this study, the ML model classifies ROIs as either positive (e.g., fungal colonies) or negative (e.g., artifacts or non-colony regions). Following classification, phenotypic traits are quantified using image processing techniques. Colony size is calculated using the *cv2.contourArea(cnt)* function, which computes the area of the contour corresponding to each fungal colony. Additionally, colony width and height are measured using the bounding box dimensions of the contour.

A robust training dataset must encompass a wide range of ROI variations, including ‘positive objects’ (mildew microcolonies) and ‘negative objects’ (artifacts or non-fungal structures). To achieve this, approximately 10,000 ROIs containing fungal colonies and an additional 8,000 ROIs with artifacts or other non-colony features were manually curated and selected, resulting in a total dataset of 18,000 images (Lueck, 2022).

For each image, the total number of fungal colonies per leaf was recorded. To account for variability in leaf size, the leaf area (in pixels) was also calculated. The final colony count was normalized by the leaf area, providing a standardized measure of fungal colonies per unit area. This approach ensures that the ML outputs (positive classifications) are directly translated into biologically relevant phenotypic traits, such as colony size, shape, and density, enabling meaningful comparisons between samples.

To evaluate the performance of the training dataset, a smaller subset containing 3,200 images per class was extracted. Both the full dataset and the smaller subset were randomly split, with 75% of the images used for training the ML models and building the classifier, and 25% used for validation and evaluation. Since convolutional neural networks (CNNs) require images of consistent dimensions, all training images were resized to 150 × 350 pixels, which represents the mean ROI size of the dataset.

#### 3.2.2 Classification using handcrafted features

Manual feature selection, also known as handcrafted feature selection, remains a widely used approach for building reliable classifiers. In some cases, it may even outperform more sophisticated methods, particularly when the objects to classify are geometrically complex (Lück et al., 2020). The success of this approach hinges on selecting ‘invariant features,’ which, in our case, are the physical and colorimetric attributes of the microcolony that remain consistent under various staining and imaging conditions. To achieve this, the ROI boundaries defined after the segmentation step were first filtered using geometrical features to reduce the presence of artifacts and non-fungal structures near positive objects (**Supplementary Table S21**).

Then, *five scale-* and *color-invariant* features Histogram of oriented Gaussians (Dalal and Triggs, 2005), *Local binary pattern* (LB) (Dong-chen and Li, 1990; Wang and He, 1990), *Haralick* (HA) (Haralick et al., 1973), *Zernike Moments* (ZM) (Tahmasbi et al., 2011), *Parameter-free threshold adjacency statistics* (PFTAS) (Coelho et al., 2010); **Supplementary Table S22**) were extracted with the *mahotas* and *scikit-image* library, and a *Random forest* classifier with 80 trees was trained with the two training sets (3,200 and 10,000 images per class).


(1) $$Accuracy=\frac{TP+TN}{TP+FP+FN+TN}$$

(2) $$Precision=\frac{TP}{TP+FP}$$

(3) $$Recall=\frac{TP}{TP+FN}$$

TP – true positive

TN – true negative

FP – false positive

FN – false negative

Equations 1–3. *Accuracy, Precision*, and *Recall* scores calculation (according to the ground truth; see the Validation chapter).

Finally, the performance of *Accuracy*, *Precision*, and *Recall* scores were calculated according to Equations 1–3.

#### 3.2.3 Convolutional neural network

We implemented a standard convolutional neural network (CNN) (**Figure 2B**) with a dropout of 0.2 and trained two training sets with different sizes (ca. 3,200 and 10,000 images per class) over 25 epochs. We used a rectified linear activation function during training and a final *SoftMax* activation function to receive the probability distribution over the classes. In addition, we used the stochastic gradient descent optimizer with a learning rate of 0.01, batch size of 32, and momentum of 0.9 to allow one training image to pass through the neural network at a time and update the weights for each layer. The final model accuracy was 97.13% (**Figure 2D**).

The CNN model consists of an input layer that accepts images resized to 150x350x3 pixels. It includes two convolutional layers, with the first employing 32 filters and the second 64 filters, both using 3x3 kernels and ReLU activation. Padding is set to “same” to preserve the spatial dimensions of the input. Following the second convolutional layer, a MaxPooling2D layer with a 2x2 pool size is applied to reduce spatial dimensions. Dropout is incorporated after each convolutional block and dense layer, with a rate of 0.2, to mitigate overfitting. The architecture includes two fully connected dense layers, the first with 1024 neurons and the second with 512 neurons, both using ReLU activation and a max-norm constraint of 3. The output layer is a dense layer with softmax activation, providing class probabilities for the two categories: positive and negative fungal structures. The model is compiled using the stochastic gradient descent (SGD) optimizer, categorical cross-entropy loss, and accuracy as the evaluation metric.

## 4 Preprocessing of the phenotypic data

Three direct phenotypes and one derivative were obtained for each genotype from detached leaf samples (**Table 1**; **Figures 1E–G**).

**Table 1:** Analyzed phenotypes.

Phenotype_ID	Phenotyping module	Phenotype	Time (hai)
48_CC	BluVision Micro	Normalized colony counts	48
48_CS	BluVision Micro	Colony area at 48 hai	48
96_CS	BluVision Micro	Colony area at 96 hai	96
0-96_AUC	BluVision Micro	The area under the growth curve 0-96 hai.	0-96

The microscopic phenotypes include normalized colony counts at 48 hours after infection (hai) with the pathogen and colony sizes at 36, 48, 56, and 96 hai. To account for the different sizes of the leaf segments, the colony counts were normalized to the area of the corresponding leaf segment. The average colony size per leaf at 48 and 96 hai was extracted from the segmented images using the *OpenCV contourArea()* function. All phenotypic values were filtered for outliers using the ROUT method with a 1% threshold.

The colony sizes at both time points were used to calculate the Area Under the growth Curve (AUC), which was also used as a phenotype in GWAS. The AUC was calculated from the 48_CS and 96_CS phenotypes according to the Equation 4.


(4) $$AUC_{0-96}=1/2*(S_{0}+S_{48})*48+1/2*(S_{48}+S_{96})*48$$

Where:

S_t_ are the BLUE of the colony size in the corresponding time points.

Since the colony area at timepoint 0 is 0 (S_0_ = 0), the simplified formula is:


$$AUC_{0-96}=24\times (2*S48+S96)$$

Equation 4. Calculation of Area Under the Curve (AUC).

To obtain robust and unbiased phenotype means for the individual genotypes from the three independent experiment repetitions, we used the Best Linear Unbiased Estimator (BLUE) (Henderson, 1975; Liu et al., 2008). BLUE was calculated with the help of the *lme4* library for R using the experiment repetitions as a random effect using the model in Equation 5.


(5) $$colony\_phenotype_{i}=\beta_{0}+\beta_{1}G_{j}+u_{GxR(i)}+\epsilon_{i}$$

Where:

*colony_phenotype_i_* is the dependent variable for the i-th observation.

*β_0_* is the intercept.

*β_1_* is the coefficient for the fixed effect *G_i_*.

*u_GxR(i)_* is the random effect associated with *GxR* for the i-th observation.

ϵ*_i_* is the residual error term for the *i*-th observation.

Equation 5. Statistical model for calculation of the Best Linear Unbiased Estimator (BLUE).

## 5 Validation

### 5.1 Validation of handcrafted feature models

One validation set of 376 colonies (Validation set I) was labeled manually as ground truth to evaluate the *Random forest* models. With the handcrafted feature (HF) *Random forest* models trained on 3,200 images per class, the local binary pattern feature reached the highest accuracy (>0.93, **Table 2**). However, the models failed colony detection on Validation set I with a false negative rate of >90% (**Table 3**). Increasing the training dataset size to 10,000 images did not improve the theoretical model accuracy of the handcrafted feature-based model, indicating that the learning curve reached a plateau (**Table 2**).

**Table 2:** Performance of the *Random Forest* model for image features trained with 3,200 or 10,000 objects per class.

Method	Training size	Precision	SD	Recall	SD	Accuracy	SD
HOG	3,200	0.8493	0.0097	0.8895	0.0110	0.8634	0.0053
LB	3,200	0.9288	0.0082	0.9421	0.0073	0.9325	0.0051
HA	3,200	0.9075	0.0100	0.9216	0.0071	0.9109	0.0056
ZM	3,200	0.7816	0.0144	0.8239	0.0075	0.7919	0.0066
PFTAS	3,200	0.8821	0.0070	0.9288	0.0082	0.9000	0.0042
HOG	10,000	0.8472	0.0081	0.8893	0.0080	0.8641	0.0059
LB	10,000	0.9346	0.0076	0.9547	0.0077	0.9429	0.0048
HA	10,000	0.9088	0.0057	0.9311	0.0059	0.9186	0.0046
ZM	10,000	0.6841	0.0116	0.7419	0.0120	0.7018	0.0064
PFTAS	10,000	0.8516	0.0082	0.8830	0.0055	0.8653	0.0056

Results represent the average of 10 independent training runs. The following edge, texture, and shape descriptors were used: Histogram of Oriented Gradients (HOG, edge), Local Binary Patterns (LB, texture), Haralick features (HA, texture), Zernike Moments (ZM, shape), and Parameter-Free Threshold Adjacency Statistics (PFTAS, texture). SD represents the standard deviation of the data.

**Table 3:** Validation set I. Handcrafted features comparison (number of colonies = 376) sensitivity and specificity in %.

Method	TP	FP	FN
HOG	25.0	3.5	75.0
LB	5.6	0.5	94.0
HA	92.0	5.0	8.0
PFTAS	88.8	10.1	11.2
PFTAS+HA	91.0	1.0	9.0

TP, True positive; FP, False positive; FN, False negative; HOG, Histogram of Oriented Gradients; LB, Local Binary Patterns; HA, Haralick features; ZM, Zernike Moments; PFTAS, Parameter-Free Threshold Adjacency Statistics.

This is usually an indication of model overfitting, resulting in a too stringent prediction or a poor capability to deal with new data. This example demonstrates how misleading the theoretical performance metrics can be if used solely without validating the model with new experimental data. Re-testing all previously built models with a new validation data set revealed the Parameter-free threshold adjacency statistics (PFTAS) and Haralick (HA) as best performing (True positives > 88%, False positives < 10%) (**Table 3**). Thus, a new model using Random forest combined with PFTAS and HA features (RF/PFTAS/HA) has significantly improved accuracy to 91% true positives and 9% false negatives, and only 1% false positives objects on the Validation set I (**Table 3**).

### 5.2 Validation of HF & CNN & HyphArea

For a direct comparison between the CNN, the handcrafted feature model, and the *HyphArea* software, we built a new Validation Set II using historical data, as the *HyphArea* system is no longer functional because of obsolete software and hardware. Although we used data generated with the *HyphArea* system, its model’s performance was significantly inferior, achieving only 25.8% true positive and 12.5% false positive rates (**Table 4**).

**Table 4:** Validation set II. A comparison of the sensitivity and accuracy of handcrafted and CNN models built on different training data sizes and *HyphArea* historical data (120 colonies).

Method	Training size	TP	FP
BluVision HC	3,200	75.89	10.01
BluVision HC	10,000	89.11	1.62
BluVision CNN 100%	3,200	85.81	9.10
BluVision CNN 100%	10,000	81.70	0.00
BluVision CNN 90%	10,000	98.31	2.53
HyphArea	NA	25.83	12.52

In contrast, the CNN and RF/PFTAS/HA models trained with the smaller training dataset (3,200 images) performed significantly better, with true positive rates of 75-85% and false positive rates of 9-10%. Training the models with the larger dataset of 10,000 images improved the true positive rate of the RF/PFTAS/HA model by 13.3%, though it decreased the true positive rate in the CNN model by 4.2%. This decrease in the CNN model may be due to chance, given the relatively small size of the validation set. However, the false positive rate for both models improved by 9%.

The CNN model offers additional flexibility by allowing adjustment of the detection threshold to meet experimental requirements. For instance, setting a threshold level of 90% significantly improved the true positive rate to 98.3% while reducing the false positive rate to only 2.5%. These results demonstrate that the CNN, supported by comprehensive validation data, provides high confidence in the phenotypic data generated by this strategy.

### 5.3 Run-time and parallel processing benchmarks

Considering the aim of a high-throughput microscopy image analysis, we optimized the algorithm for run-time per image. Besides other improvements, using numerical *Python* libraries, which allow efficient numerical calculations on multi-dimensional arrays, and parallelizing the processes with the *joblib* library (*Python*) led to a significant speed gain. As a result, *BluVision Micro* performed up to 30 times faster than the previous *HyphArea* software in analyzing pyramid images of average size 30,000 x 25,000 pixels. On an Intel^®^ Core™ i7-9700 CPU 3.00 GHz with 64-bit Windows 10 operating system and *NVIDIA TITAN X GPU* support, the software run time takes about 60 seconds per slide containing two images of size 30,000 x 25,000 pixels, which is 3-5 faster than the image acquisition time, thus allowing real-time analysis.

### 5.4 Feature visualization

Visualizing the CNN predictions becomes crucial because of the increasing demand for transparency of the artificial intelligence prediction models. However, the availability of visualization options was limited until recently, when several such tools were developed. To examine the *BluVision Micro* CNN model’s prediction and facilitate debugging, we used *Keras Visualization Toolkit* (Zhou et al., 2015) to generate heatmap images to visualize the *Class activatio n maps* for the fungal structures. The resulting heatmaps correctly represented the Area covered by the fungal microcolonies (**Figure 2C**). Examples using further visualization tools are present in the **Supplementary Data**.

## 6 Image analysis pipeline description

After selecting and tuning the separate components of the system, they were assembled into an image analysis pipeline. The image data from the CZI files is loaded into memory for processing. This step is essential to accelerate accessing and manipulating the image content, but it requires high installed memory. The meta-information about all object dimensions, resolution, and other specific characteristics is retrieved from the CZI files. Notably, the CZI format often contains multiple regions requiring separate processing. Next, a stacked image is generated from the z-stack using the *Minimum-intensity projection* method. The *Minimum-intensity projection* method involves creating a single image from multiple layers (z-stack) by taking the minimum intensity projection, which helps in reducing noise and enhancing the features of interest. A binary image is then created by converting the *RGB* image into *YQ1Q2* color space and utilizing only the Q2 channel. This conversion helps in isolating specific features of the image, making it easier to identify regions of interest and reducing the background noise. The process continues with extracting all contour objects as potential regions of interest (ROIs) using *OpenCV*. *OpenCV’*s contour detection algorithms are employed to identify the boundaries of objects within the binary image. Simple geometric filters are applied to remove unwanted objects that are too small or too large. These filters help refine the set of potential ROIs by eliminating irrelevant or extraneous objects based on their size and shape. Finally, the objects are classified using a CNN model. This comprehensive pipeline ensures that the images are processed efficiently in parallel, with the relevant regions identified and classified accurately. The results, which include features of each detected colony, leaf size, and summary statistics (mean, median colony size and count per leaf, standard deviation, normalized colony counts, etc.), are exported as standard comma-separated values (CSV) files.

## 7 Software installation and usage

The *BluVision Micro* software installation was performed using *Anaconda* to create a virtual environment with *Python* version 3.6 or higher. *Anaconda* was downloaded and installed from its official website (https://www.anaconda.com/). The *BluVision Micro GitHub* repository was cloned to the local machine, and all necessary dependencies were installed. Comprehensive installation and usage instructions are available in the *GitHub* repository (refer to Data Availability).

## 8 Transferability to the wheat powdery mildew system

The robustness of the developed software is demonstrated by its successful application to the wheat-powdery mildew system without the need for major adjustments. The segmentation algorithm, designed to distinguish PM hyphae from leaf background, proved to be effective for wheat leaves as well. Adaptive thresholding and morphological operations used for barley images were directly applicable to wheat, indicating the algorithm’s versatility in handling different leaf structures and textures. Feature extraction for wheat leaves involved the same shape and texture features used for barley. To evaluate the transferability of the model to a wheat powdery mildew system, we generated a wheat dataset comprising ten Kanzler wheat leaves with 1,123 hyphal colonies. This dataset posed a particular challenge due to the presence of large trichomes—leaf structures that are either absent or significantly less pronounced in barley—and suboptimal staining. Despite these complexities, the model, originally trained on barley, achieved a true positive rate of 92% and a false positive rate of just 1% (**Supplementary Table S24**). These results suggest that while the model may miss some colonies in this more complex dataset from a different species, its ability to maintain a very low false positive rate underscores its robustness.

## 9 Morphometric traits

**Figure 3:**
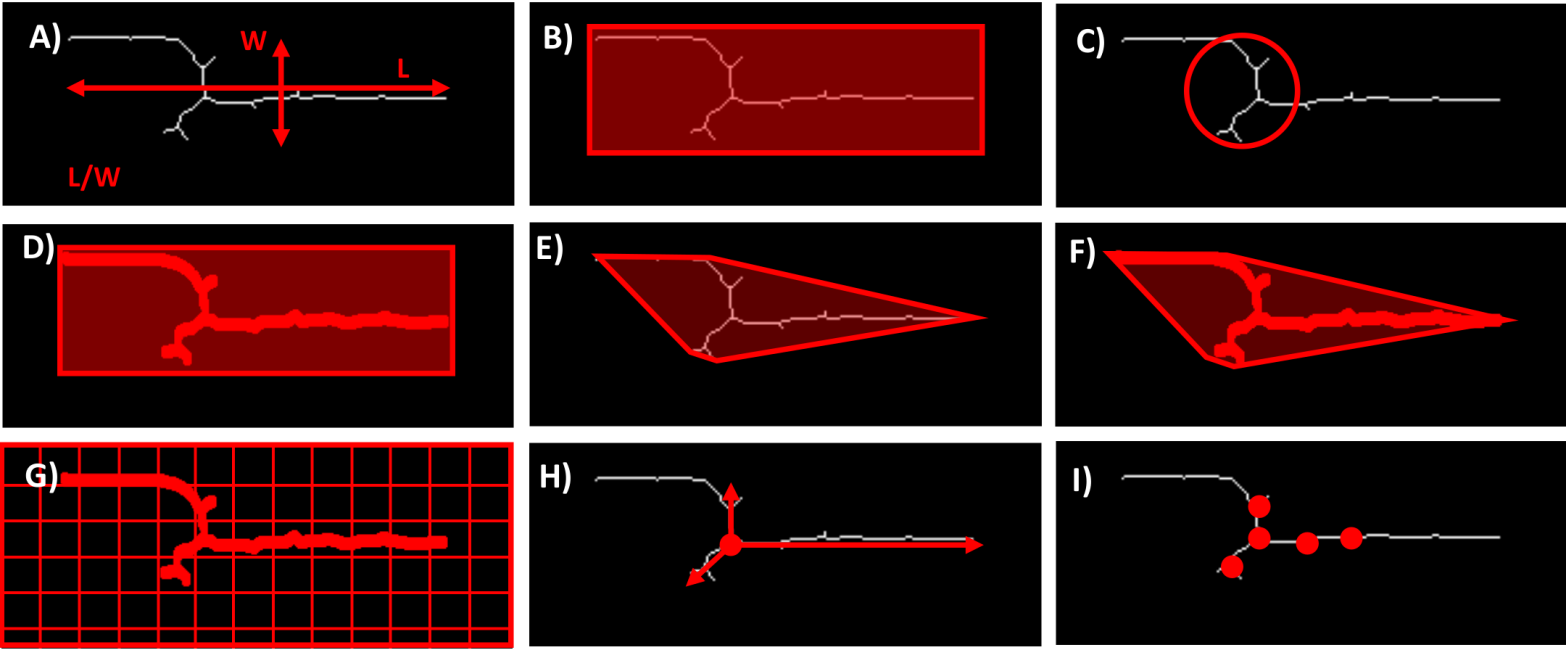
Examples of possible morphometric traits. **(A)** Aspect ratio - the ratio of the length to the width of the hyphae. **(B)** Bounding box area. **(C)** Circularity **-** quantifies how close the shape of the hyphae is to a perfect circle. **(D)** Extent - is the ratio of the Area occupied by the hyphae to the Area of the bounding box that encloses them. **(E)** Convex area - refers to the Area of the smallest convex shape that can enclose the hyphae. **(F)** Solidity - measures the proportion of the convex area that is actually occupied by the hyphae. **(G)** Hyphal density - refers to the amount of hyphal material present within a given area or volume. **(H)** Radial growth rate - measures the speed at which the hyphae expand outward from the point of origin. **(I)** Number of branch points **-** indicates the frequency of hyphal branching.

In addition to directly measuring the number and size of the colonies, several morphometric traits can be automatically derived from the image data. These traits provide a more comprehensive understanding of the hyphal growth patterns and their interactions with the host. Some of the key morphometric traits are shown in **Figure 3**.

These morphometric traits offer a detailed characterization of the fungal growth dynamics. Depending on the purpose of the analysis, integrating several of these phenotypes can provide a more nuanced and complete picture of plant-fungal interactions. This comprehensive approach enhances the ability to study fungal pathogenicity, host resistance mechanisms, and the overall impact of fungal colonization on plant health.

## 10 Limitations

While the software application for detecting and classifying powdery mildew on barley and wheat leaves demonstrates high accuracy and robustness, it faces challenges in detecting hyphae older than 72 hours post-inoculation due to the intertwining growth of hyphae. The limit can be extended to 96 hours by using lower inoculation spore density, but the segmentation of individual colonies remains challenging at later time points.

## 11 Downstream applications and results

### 11.1 Genome-wide association scans using microscopic infection phenotypes

To showcase the system’s utility in phenotyping microscopic infection traits, we conducted a genome-wide association study (GWAS) on barley powdery mildew.

The experiment design (**Figures 1E–G**) allowed the quantification of multiple phenotypes (**Table 1**) from a single leaf. The precise phenotypic data was combined with the dense SNP data (949 174 quality SNPs) for GWAS for resistance-associated markers.

Since the study aims to provide proof of concept and application examples, the number of tested genotypes was limited to 196. This number is on the lower end for detecting significant marker-trait associations (MTAs) in genetically diverse materials. To provide a broader view of the genetic landscape and increase the chances of discovering causative genes, proximal MTAs were aggregated into MTA blocks (MTAB) with a minimum size of 0.5 Mb or larger if the MTAs were in linkage disequilibrium.

**Figure 4:**
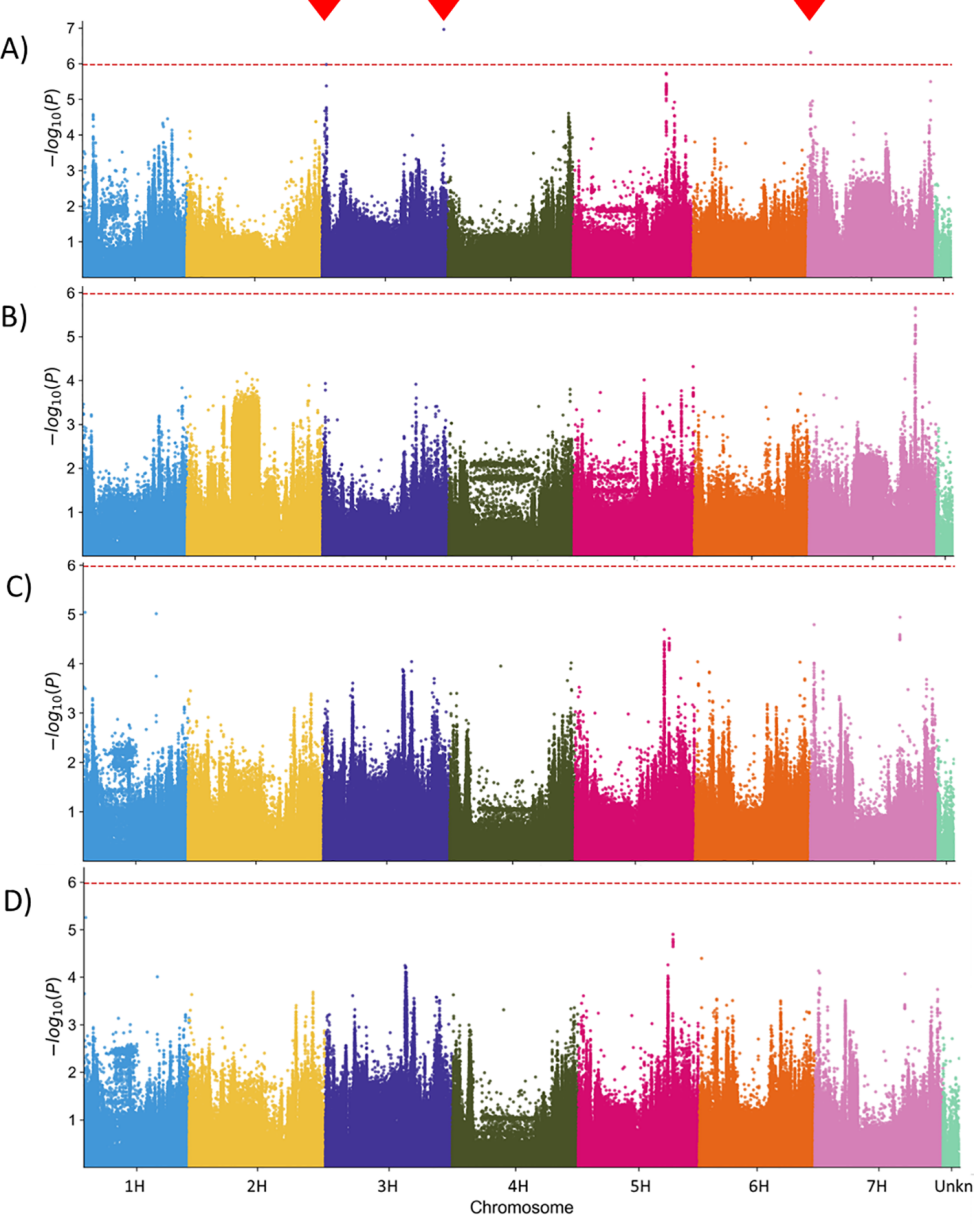
Manhattan plot of the [-log_10_] transformed p-values for statistical significance of the marker-trait associations in Genome-Wide Association Scan (GWAS). **(A)** Genomic regions associated with the normalized Bgh colony counts at 48 hai (48_CC). **(B-D)** No genomic region was significantly associated with the mean Bgh colony size at 48 hours **(B)** and 96 hours **(C)** after inoculation, as well as with the Area under the growth curve (0-96AUC) **(D)** phenotype. Red dashed line – suggestive threshold after corrections for multiple testing. Red triangles indicate the locations of statistically significant associated markers.

As expected, the 48 hai colony counts delivered the most significant MTAs (**Figure 4A**) since the penetration resistance against powdery mildew fungus, which effectively reduces the number of successful infection events, is widespread in barley. However, the MTAs reached only the suggestive threshold of -log_10_ P value of 6, not the significance threshold of -log_10_ P >8, which was relatively high because of the multiple test correction for the large number of SNPs included in the analysis (~1,000,000). Significant associations were detected on both ends of chromosome 3H and at the beginning of chromosome 7H (**Figure 4A**). The three MTABs contained a total of 53 gene annotations (see the **Supplementary Table S5**). Some genes with a clear link to the plant pathogen defense are listed in **Table 5**. The complete list is available in the **Supplementary Material** (**Supplementary Table S6**).

**Table 5:** A list of genes in MTABs contains markers significantly associated with the colony count at 48 hai phenotype (48_CC) from protein families well-known to be involved in plant-pathogen interactions.

MTA_Block	Chr.	Gene ID	Gene description	Putative function	References
48_CC_MTAB_13	chr3H	HORVU.MOREX.r3.3HG0321190	F-box protein	Protein degradation	(Marino et al., 2012)
48_CC_MTAB_13	chr3H	HORVU.MOREX.r3.3HG0321210	Receptor kinase-like protein	Pathogen sensing	(Yang et al., 2012)
48_CC_MTAB_13	chr3H	HORVU.MOREX.r3.3HG0321270	Arf GTPase activating protein	Membrane trafficking	(Rivero et al., 2019)
48_CC_MTAB_29	chr7H	HORVU.MOREX.r3.7HG0642520	Peroxidase	Defense	(Dos Santos and Franco, 2023)
48_CC_MTAB_29	chr7H	HORVU.MOREX.r3.7HG0642550	Peroxisomal membrane protein	Peroxisome targeting	(Pan et al., 2020)
48_CC_MTAB_29	chr7H	HORVU.MOREX.r3.7HG0642600	Receptor-like protein kinase	Pathogen sensing	(Yang et al., 2012)
48_CC_MTAB_29	chr7H	HORVU.MOREX.r3.7HG0642690	ABA-responsive binding factor	ABA signaling	(Fan et al., 2009)
48_CC_MTAB_29	chr7H	HORVU.MOREX.r3.7HG0642810	RNase P Rpr2/Rpp21 subunit	RNA processing	(Woloshen et al., 2011)
48_CC_MTAB_29	chr7H	HORVU.MOREX.r3.7HG0642820	RNase P Rpr2/Rpp21 subunit	RNA processing	(Woloshen et al., 2011)
48_CC_MTAB_10	chr3H	HORVU.MOREX.r3.3HG0229300	Germin-like protein	Disease resistance	(Govindan et al., 2024)
48_CC_MTAB_10	chr3H	HORVU.MOREX.r3.3HG0229310	Receptor-like protein kinase	Pathogen sensing	(Yang et al., 2012)
48_CC_MTAB_10	chr3H	HORVU.MOREX.r3.3HG0229320	Receptor-like protein kinase	Pathogen sensing	(Yang et al., 2012)
48_CC_MTAB_10	chr3H	HORVU.MOREX.r3.3HG0229400	Receptor-like protein kinase	Pathogen sensing	(Yang et al., 2012)
48_CC_MTAB_10	chr3H	HORVU.MOREX.r3.3HG0229360	F-box family protein	Protein degradation	(Marino et al., 2012)
48_CC_MTAB_10	chr3H	HORVU.MOREX.r3.3HG0229400	Receptor-like protein kinase	Pathogen sensing	(Yang et al., 2012)
48_CC_MTAB_10	chr3H	HORVU.MOREX.r3.3HG0229420	Receptor-like protein kinase	Pathogen sensing	(Yang et al., 2012)
48_CC_MTAB_10	chr3H	HORVU.MOREX.r3.3HG0229450	RNA exonuclease 4	RNA processing	(Maksimov et al., 2021)
48_CC_MTAB_10	chr3H	HORVU.MOREX.r3.3HG0229460	RNA exonuclease 4	RNA processing	(Maksimov et al., 2021)
48_CC_MTAB_10	chr3H	HORVU.MOREX.r3.3HG0229470	RNA exonuclease 4	RNA processing	(Maksimov et al., 2021)
48_CC_MTAB_10	chr3H	HORVU.MOREX.r3.3HG0229480	Actin/actin-like family protein	Cytoskeleton	(Li and Day, 2018)
48_CC_MTAB_10	chr3H	HORVU.MOREX.r3.3HG0229520	E3 ubiquitin-protein ligase	Protein degradation	(Marino et al., 2012)
48_CC_MTAB_10	chr3H	HORVU.MOREX.r3.3HG0229610	Actin cross-linking protein	Cytoskeleton	(Li and Day, 2018)

The Morex V2 gene identifiers were remapped to the latest Morex V3 assembly using nucleotide BLAST.

**Figure 5:**
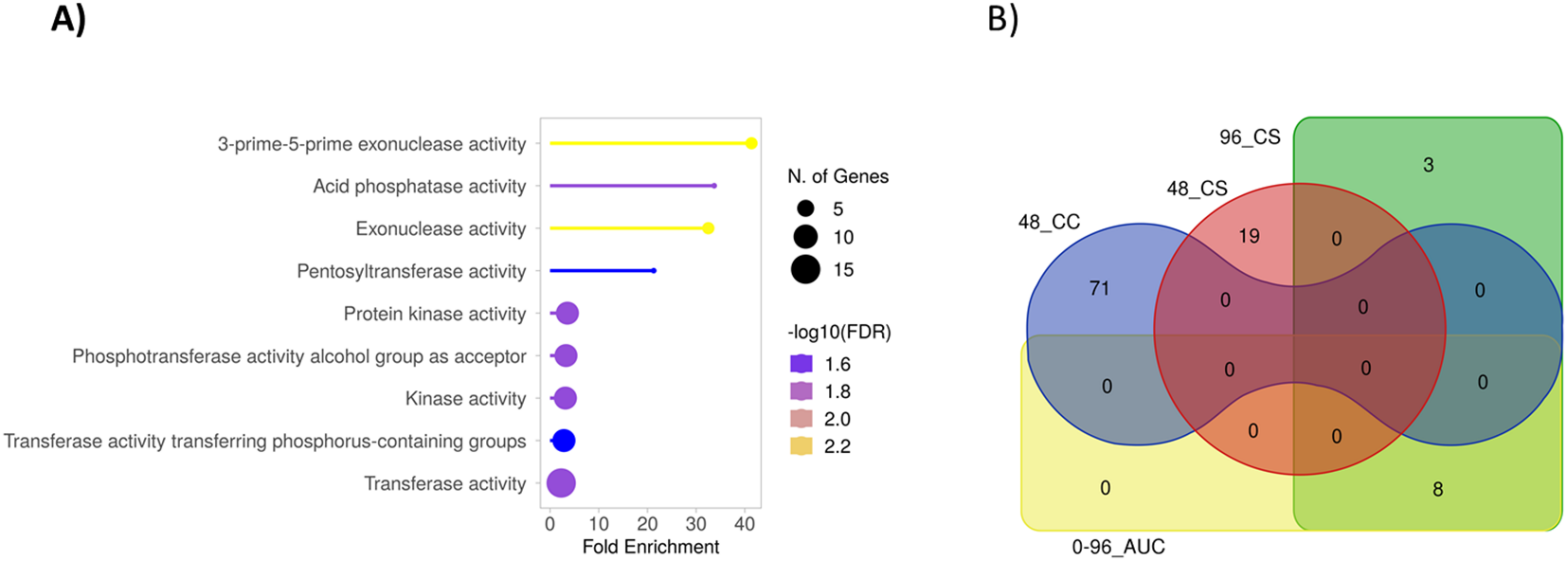
**(A)** Protein domain overrepresentation analysis for the genes located in the MTABs with -log_10_ P values >6 on chromosome 3H and 7H (52 genes) using the *Morex* V2 gene ID against the hvtritex_eg_gene gene database (*Morex* V2 TRITEX assembly) and GO Molecular Functions pathway database [ShinyGO online tool (Ge et al., 2020)]. **(B)** A Venn diagram for the intersections of the gene annotations in MTABs associated with the different phenotypes. The statistical significance was relaxed to -log P value >5 to broaden the range of included genes.

The protein domain overrepresentation analysis of the genes in these MTABs revealed significant enrichment of exonucleases, phosphatases, protein kinases, and transferases (**Figure 5A**). Notably, at least seven RLKs are located in the MTABs. The relatively high number of RLKs is striking, considering that they are a prominent class of plant immune receptors commonly implicated in disease resistance.

The *BluVision Micro* platform provides the possibility to measure precisely, and in high-throughput, the area of the secondary hyphae of the powdery mildew colonies. This opens new phenotyping options, which are hardly possible with manual microscopy. For instance, measuring the colony size at a specific time point after inoculation may reveal plant defense mechanisms that rely on retarding the pathogen growth, e.g., cutting the nutrient support for the fungus or late activation of cell death mechanisms.

The colony size-based phenotypes (*48_CS* and *96_CS*) (**Figure 4B**, respectively **Figure 4C**) did not deliver significant MTAs in this genotype population. This is not unexpected because a natural resistance in barley based on microscopically-measurable colony growth retardation, to our best knowledge, is not yet described in the literature, not at last because of the lack of screening methods. However, such valuable phenotype likely exists, and a systematic screen of diverse plant genotypes may help discover it. The domain enrichment analysis for the genes located in MTABs with -log_10_ P values >5 did not reveal a significant overrepresentation of specific classes of genes.

**Figure 6:**
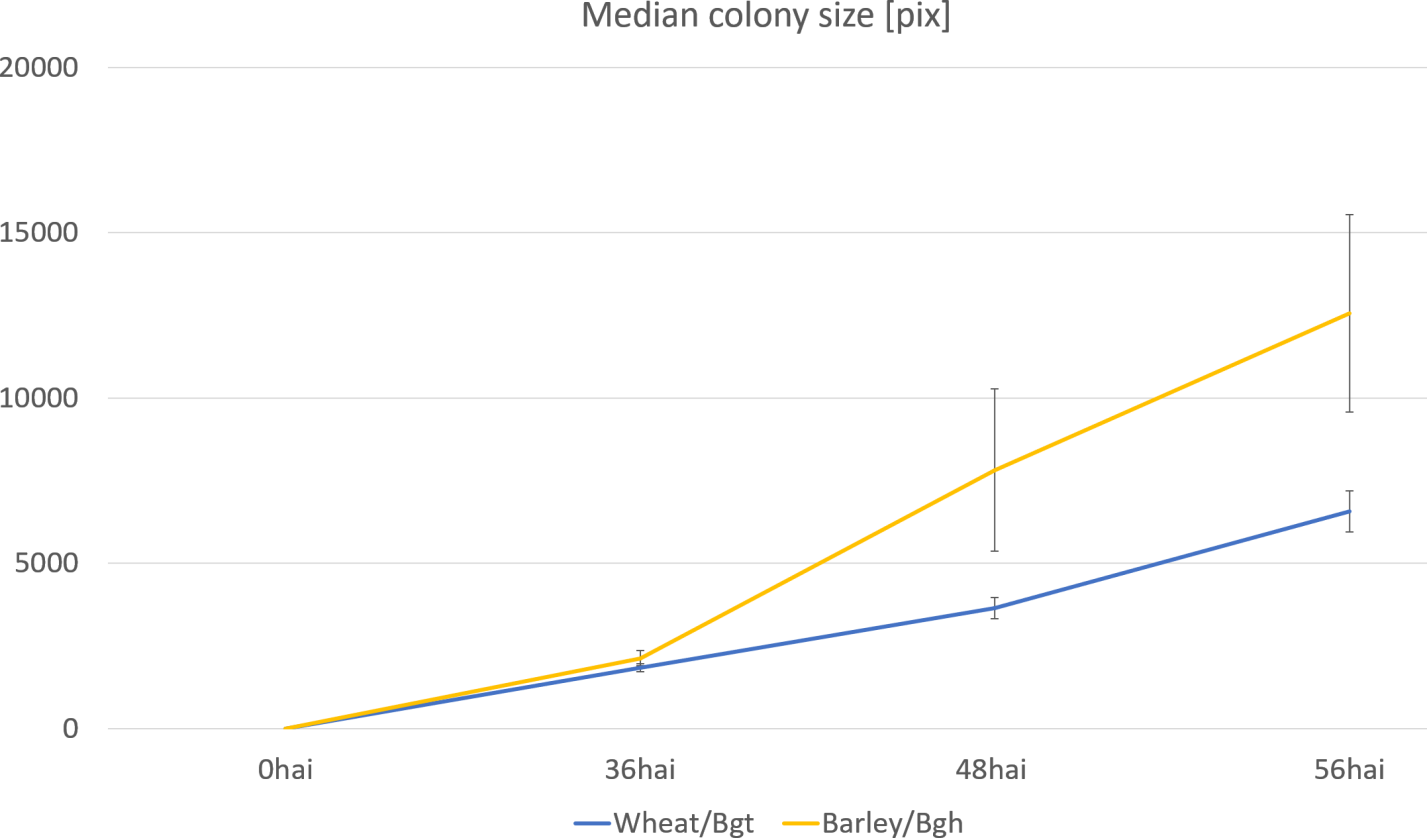
Growth curve of two adapted powdery mildew species on wheat and barley, respectively. The mean colony sizes were estimated on samples collected 36, 48, and 56 hours after inoculation with Bgh or Bgt. SD, Standard deviation of the data.

We utilized the BLUEs for colony sizes at 48 and 96 hai across 196 barley genotypes to construct genotype-specific growth curves, using the Area Under the Curve (AUC) as the phenotype for GWAS (**Figure 4D**). While none of the MTAs for colony size phenotypes reached the suggestive threshold, this novel phenotyping method holds promise for identifying plant resistance mechanisms that influence pathogen growth rates. Additionally, it serves as a valuable tool for comparing the fitness of different pathogen races or pathogens through growth curve analysis (**Figure 6**).

To estimate the diversity of genes located in MTABs associated with various phenotypes, we performed an intersection analysis, illustrated by the Venn diagram in **Figure 5B**. Interestingly, aside from the related 96_CS and 0-96_AUC phenotypes, which share eight common genes, no overlapping genes were found to be associated with the different phenotypes.

As already mentioned, the high number of SNPs increases the levels of statistical significance due to multiple test corrections. However, several other MTABs contain markers with significance approaching the thresholds, which may still harbor valuable genes. Therefore, we provide a comprehensive list of all genes in MTABs with markers associated with the phenotype at a significance level of -log_10_ P value >4, as well as all individual MTAs with a -log_10_ P value >4. These details are included in the **Supplementary Material** (**Supplementary Tables S6**-**S17**).

## 12 Discussion

The need for automated microscopic phenotyping of plant-pathogen interactions became apparent with increasing the number of available genetic and genomics resources and the pursuit of finding novel genes putatively involved in the complex phenomenon of disease resistance.

The *HyphArea* tool has been instrumental in advancing plant-pathogen interaction phenotyping by enabling the detection and quantification of secondary hyphae of *B. graminis*. While its pioneering approach has opened avenues for exploring novel phenotypes, such as fungal hyphal area, its application has revealed certain limitations in the context of large-scale phenotyping. Variability in sample quality has often hindered the reproducibility of its high sensitivity and specificity levels reported in earlier studies. Moreover, the practical challenges associated with high-throughput use, particularly the inefficiencies in data handling due to the large number of TIFF files generated and the considerable processing time required. These constraints underline the need for further advancements in phenotyping tools to better accommodate the demands of large-scale, high-throughput workflows.

Benefiting from the accumulated experience and using newer high-throughput automated microscopy and software techniques, we have developed a new system for microscopy-based phenotyping. We decided to opt for a modular, machine learning-based software that works directly with different image data types, including complex pyramid files and multimodal images, and it is easily adaptable and extendable with modules for additional phenotypes.

Different machine learning (ML) approaches were tested and evaluated. Handcrafted features-based ML models, if chosen correctly, can provide acceptable performance in cases where only small (< 5,000 images per class) training sets are available. Using more training data for the handcrafted features approach does not further increase the performance, showing that we have reached the methods’ limits in this case. For higher accuracy and more extensive training sets (> 5,000 images per class), we recommend using a CNN, whose significant advantage is extracting the probability for each class and using it as a parameter for predictions.

The newly developed *BluVision Micro* system provides precise microscopic phenotyping information for various large-scale studies, including screening Genebank material, crossing populations, mutant collections, and breeding material for both host and pathogen sides. This study used the system to screen genetically diverse barley collection for interaction phenotypes with powdery mildew fungi. The system demonstrated accurate, sensitive, and reproducible results, which we used to scan for marker-trait associations in the barley genome, identifying several loci potentially associated with the traits of interest. Additionally, the system enables high-throughput studies of previously laborious phenotypes, such as precise colony area measurement and the scoring of pre- and post-haustorial defense reactions. With the use of other dedicated modules, the *BluVision* platform can be employed for fluorescence microscopy or to detect fungal haustoria in reporter gene (GUS) expressing cells. This capability facilitates high-throughput transfection assays for disease resistance-related genes, significantly enhancing research efficiency and accuracy.

The open-source software system supports the development of specific modules for various microscopic phenotypes. Developed using object-oriented principles, the software framework allows for easy extension to accommodate additional pathogens and new modules. It is designed to be adaptable to various file formats, including DICOM standards and single images, enhancing its versatility beyond its initial application.

The *BluVision Micro* system was used for phenotyping 196 barley genotypes infected with barley powdery mildew. Three direct phenotypes and one derivative phenotype were quantified, with the number of developed microcolonies providing the most informative results. Three major marker-trait association blocks (MTABs) were localized on chromosomes 3H (2 MTABs) and 7H. A total of 53 genes were annotated in these regions. Protein domain overrepresentation analysis revealed significant enrichment of exonucleases, phosphatases, protein kinases (including seven receptor-like kinases), and transferases (**Figure 5A**). Many of these genes belong to protein families, which are well known to be involved in plant defense reactions. Exonucleases play a crucial role in plant disease resistance by participating in key processes such as DNA repair and maintenance (Britt, 1999), RNA silencing, programmed cell death (PCD) (Coll et al., 2011), and generation of signaling molecules (Voinnet, 2009). Phosphatases also play a significant role in plant defense, contributing to signal transduction (Schweighofer et al., 2004) and PCD (Coll et al., 2011).

However, probably the most interesting was a significant enrichment of receptor-like kinases (RLKs), which are major players in plant immunity. Besides functioning as canonical receptors for pathogen-associated molecular patterns (PAMPs) (Zipfel, 2008; Boller and Felix, 2009), RLKs are involved in signal transduction, production of reactive oxygen species (ROS) (Li et al., 2014), PCD (Coll et al., 2011), and pathogen-induced endocytosis (Mbengue et al., 2016).

These findings provide indications that these genomic regions may be associated with disease-resistance phenotypes. Furthermore, they suggest that the *BluVision Micro* system has the potential to capture meaningful phenotypic data, which could support the discovery of novel genes involved in disease resistance. The observed enrichment of genes related to key defense mechanisms underscores the potential significance of these MTABs as regions of interest for breeding disease-resistant barley varieties.

In conclusion, we have developed an open-source, extendable, high-throughput automated system for the analysis of microscopic phenotypes. We validated the system’s performance in disease resistance screens of genetically diverse barley material and demonstrated that the phenotypic data could be used for Genome-Wide Association Scans (GWAS), discovering several resistance-associated loci.

## Data Availability

The datasets presented in this study can be found in online repositories. The names of the repository/repositories and accession number(s) can be found in the article/**Supplementary Material**.
