# Supplementary material for: Deep phenotyping platform for microscopic plant-pathogen interactions
Source: Front Plant Sci. 2025 Feb 3;16:1462694. doi: 10.3389/fpls.2025.1462694 (PMC11832026; doi:10.3389/fpls.2025.1462694)
Supplement: Supplementary file 1 [file DataSheet1.pdf]

Supplement 1: BluVision Micro workflow

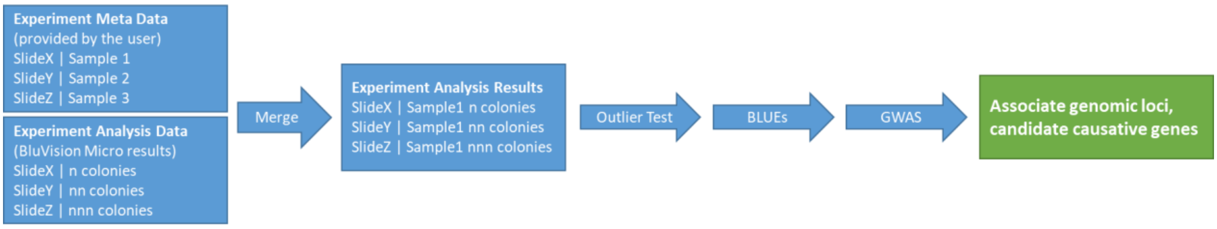

1. The experimental workflow begins with the systematic collection of metadata, including key details such as accession numbers, inoculation density, and species. This metadata serves as the foundational reference for subsequent analysis.

2. Following metadata acquisition, slides are scanned and analyzed using the *BluVision Micro* software.

3. The results obtained from this analysis are then merged with the metadata, creating a comprehensive dataset that links experimental conditions with image-derived traits.

4. An optional step in the analysis involves applying the ROUT outlier test with a threshold of 0.01 to identify and exclude extreme data points that could potentially bias downstream analyses.

5. Subsequently, Best Linear Unbiased Estimates (BLUEs) are calculated. This step is particularly important when combining data from multiple experiments conducted under varying environmental or experimental conditions. By accounting for these variations, BLUEs standardize the trait values, enabling meaningful comparisons and reducing confounding effects that might otherwise obscure the genetic signal.

6. Finally, genome-wide association studies (GWAS) are performed using the GWAStic software (Lück et al., 2024, Bioinformatics Advances), to identify genetic loci associated with the traits of interest. The integration of metadata, image-based phenotyping, and statistical normalization ensures that the GWAS results are both robust and biologically meaningful, providing insights into the genetic basis of the studied traits.

Supplement 2: CNN visualization

To improve interpretability and confidence in the model's classifications, we incorporated visualization tools as follows:

Grad-CAM: We used Grad-CAM (Gradient-weighted Class Activation Mapping) to visualize the regions of input images that contribute most to the classification decisions. For this, we applied Grad-CAM to two representative fungal colony samples from the dataset and highlighted the key features identified by the model. These visualizations demonstrate that the model focuses on morphological structures like hyphal growth patterns when identifying fungal colonies.

Feature Map Extraction: We saved the feature maps from all convolutional layers for the same two colony samples. These feature maps provide insights into how the model progressively extracts and refines features at different layers, from edges and textures in early layers to complex patterns in deeper layers.

By incorporating these tools, we not only clarified the inner workings of our CNN but also ensured that the classifications align with biologically relevant features, thereby increasing confidence in the model's outputs.

The visualization scripts and extracted maps have been made available in our project repository for reproducibility.

Gradient-weighted Class Activation Mapping of a positively classified hyphal colony

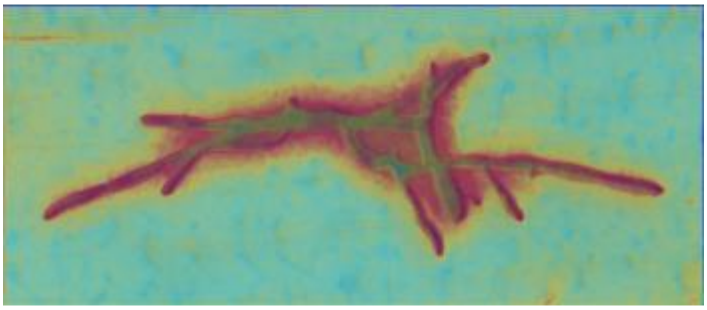

The feature map in this image highlights model activations with strong red regions indicating high activation, particularly concentrated on the main branching structure, suggesting the model focuses on the central part for its prediction. The yellow-green gradient represents intermediate activations where features are less prominent but still relevant, while the blue background signifies low activation areas with no significant features. The spread of red-hot activations across the branching structure suggests the model considers a broader region relevant, likely due to diffuse feature detection or broader receptive fields in deeper convolutional layers. Overall, this indicates that the model correctly identifies the target object while minimizing focus on background noise.

Feature Map 2 of Conv2D layer 1 of a positively classified hyphal colony

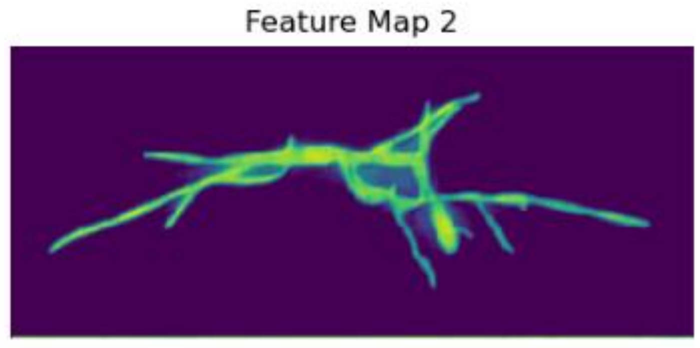

The feature map, using a viridis-style colormap, highlights a thin, branching structure resembling roots, fungal hyphae, or similar elongated biological forms, with bright green/yellow areas indicating strong activations and dark purple/blue areas representing low or no activations. The filter detects elongated, tubular patterns and edges, effectively focusing on the structure while suppressing background noise, suggesting it is sensitive to line-like features. The clear, interpretable shape of the activation indicates that this map likely originates from an early convolutional layer, which typically captures low-level features such as edges and simple shapes.

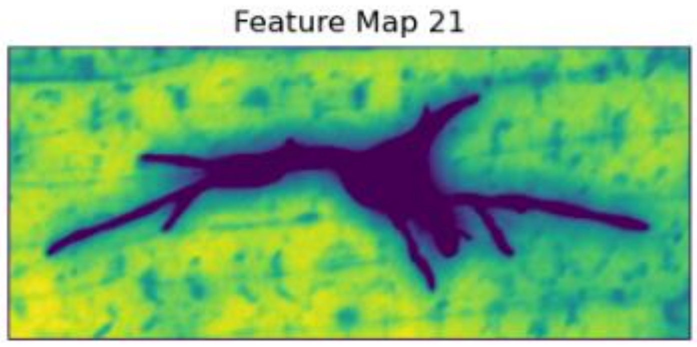

Feature Map 21 of Conv2D layer 1 of a positively classified hyphal colony

This feature map, using the same viridis colormap, highlights both the main branching structure and background activations, with bright yellow and green areas representing high activations and dark purple and blue areas showing low activations. Unlike earlier maps, the activations here are more diffused, with noticeable horizontal bands in the background, suggesting sensitivity to textures or gradients. While the main branching structure remains visible, it appears less sharply defined, indicating the filter focuses on broader, lower-frequency patterns rather than fine details. This feature map likely captures textures and spatial regions, combining earlier edge-detection outputs to abstract more complex features.

Example report 1

This example report contains the original image, the Grad-CAM visualization, and the 32 feature maps from the first convolutional layer. The original image provides the input for analysis, while the Grad-CAM highlights the key regions influencing the model's prediction. The 32 feature maps reveal the outputs of individual filters in the first Conv2D layer, showcasing various activations that detect edges, textures, and patterns within the image.

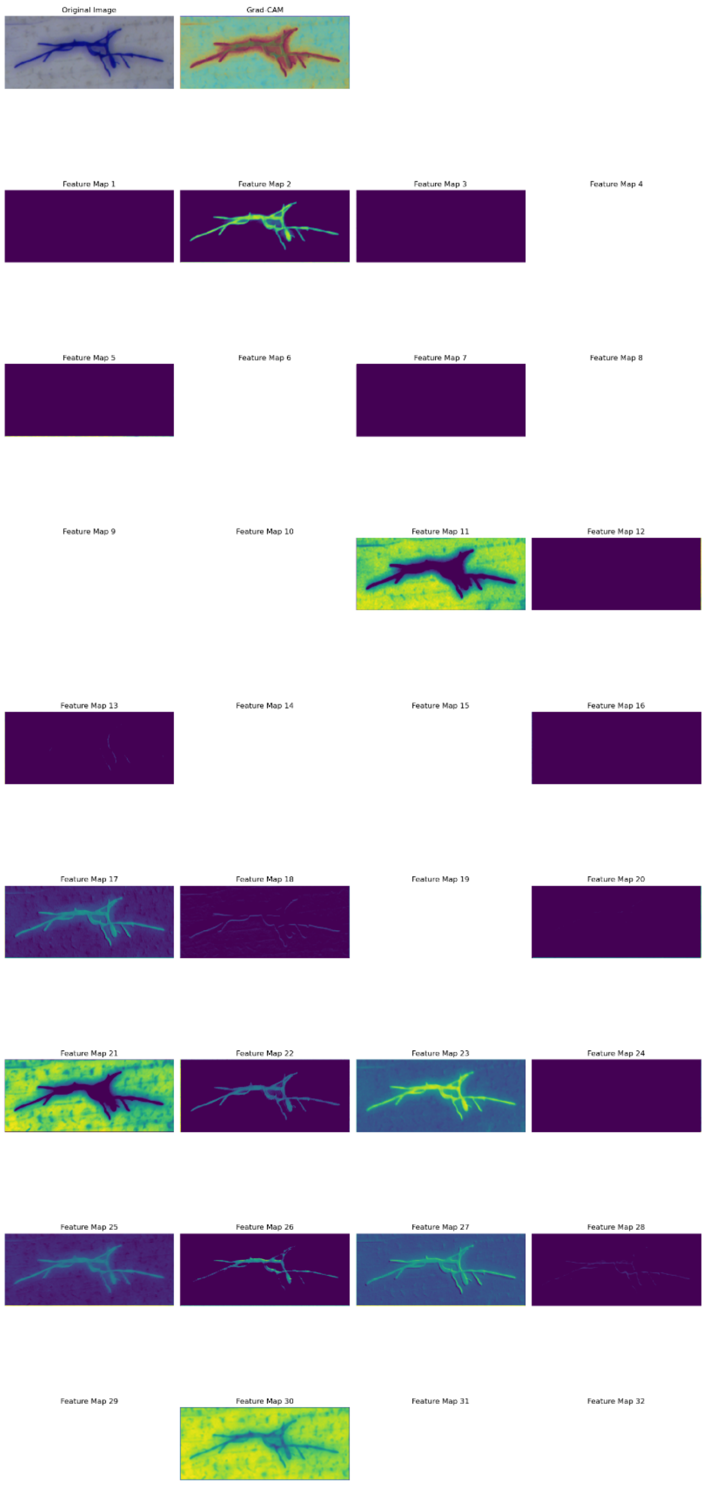

Example report 2

This example report shows another positively classified colony, highlighting the activations that led to the correct prediction. In contrast, files report_4.png and report_3.png display negatively classified examples, where the activations likely differ significantly, focusing on irrelevant regions or failing to capture the key features of the structure.

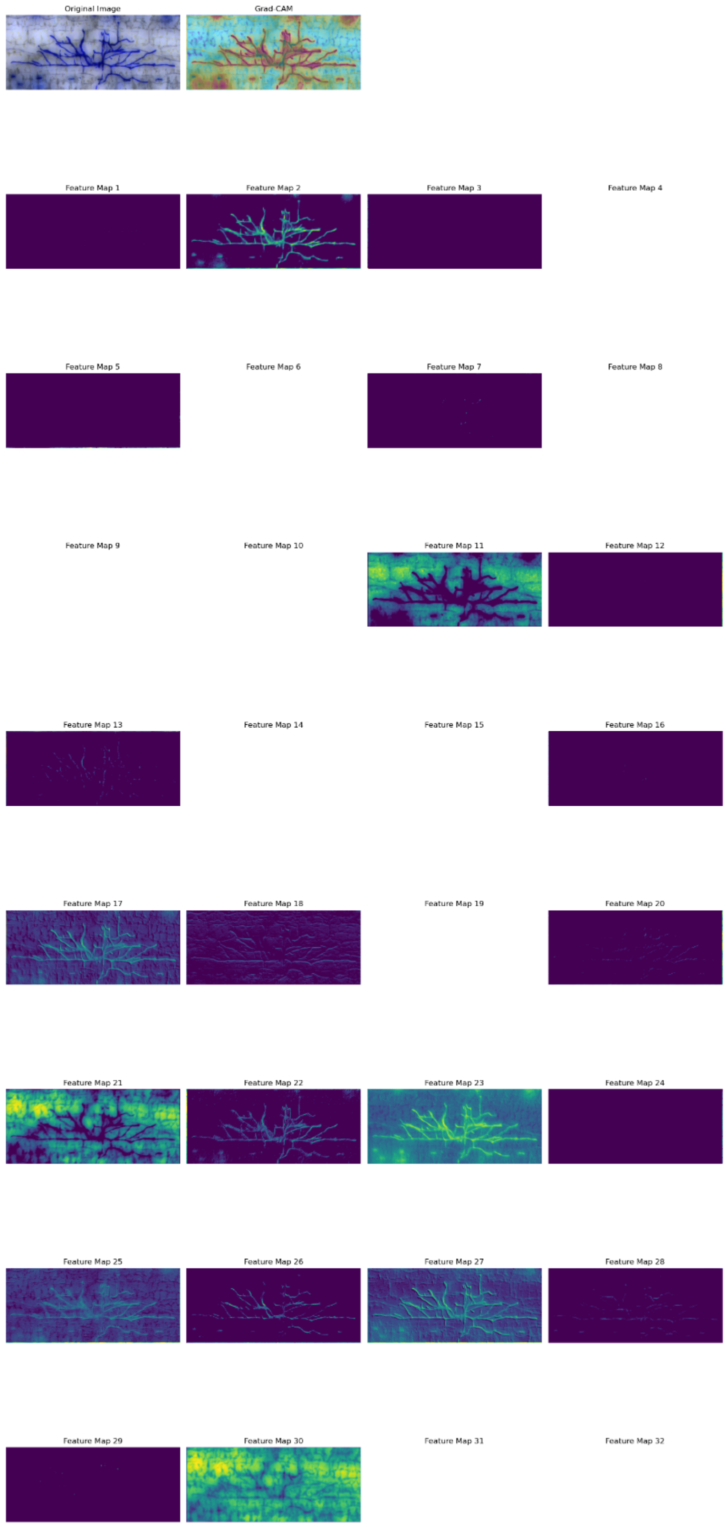

Supplement 3: Phenotyping using the Macrobot platform (BluVisoon Macro) on the same 200 barley genotypes described in the manuscript.

Barley often exhibits a genotype-specific issue known as physiological necrosis. This condition causes certain genotypes to develop necrotic spots on the leaves, preventing biotrophic pathogens like powdery mildew from growing and making these genotypes appear “resistant.” This phenomenon typically occurs 4–5 days after leaf cutting. Since the Macrobot phenotyping (Lück et al. 2020) occurs after disease symptoms become visible, usually 5–6 days post-infection, the data on barley powdery mildew from the Macrobot is less reliable compared to wheat or other grass species. Nevertheless, we conducted such an analysis, and the results are provided below.

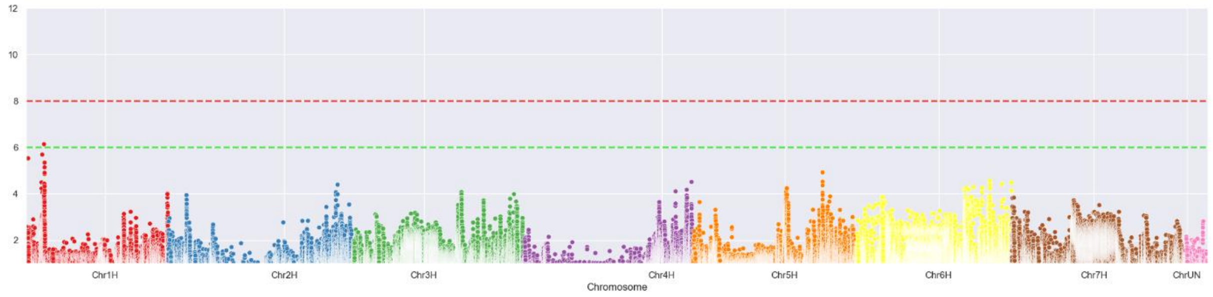

Genomic regions associated with the size of the visibly infected area of the leaf, seven days after Bgh inoculation. Manhattan plot of the [-log_{10}] transformed p-values for the Bgh_168hai_area phenotype. Green dashed line – suggestive threshold, red dashed line – significance threshold.

Lück, S., et al. (2020), '"Macrobot": An Automated Segmentation-Based System for Powdery Mildew Disease Quantification', *Plant Phenomics,* 2020, 5839856.
